# Victim and Offender Self-Reports of Alcohol Involvement in Crime

**Published:** 2001

**Authors:** Lawrence A. Greenfeld, Maureen A. Henneberg

**Affiliations:** Lawrence A. Greenfeld, M.S., is the deputy director and Maureen A. Henneberg, M.P.A., is the associate director of the Bureau of Justice Statistics, U.S. Department of Justice, Washington, D.C.

**Keywords:** AODR (alcohol or other drug [AOD] related) crime, AODR violence, offender, self-report, victim of crime, probation, jail inmate, AOD use pattern, trend, survey

## Abstract

Research suggests that a decreasing share of violent crime is attributable to offenders who had been drinking alcoholic beverages. Surveys of victims indicate that the rate of alcohol-involved violent crimes (i.e., crimes in which the perpetrators had been drinking, as perceived by the victims) decreased 34 percent from 1993 to 1998, whereas the rate of non-alcohol-involved violence decreased 22 percent. Surveys of some offenders also suggest that alcohol’s role in violence is decreasing. The decrease in alcohol-involved violence is consistent with declines in other measures of alcohol use and misuse, including per capita alcohol consumption and alcohol involvement in traffic crashes. In contrast, violent offenders in State prisons are increasingly likely to report having used alcohol before committing their offenses, possibly illustrating the effect of more severe sanctions for alcohol-involved offenses.

Violent crime in the United States decreased at an unprecedented rate during the 1990s. According to victim surveys, approximately 23 percent fewer violent crimes occurred in 1998 than in 1993. Similarly, data from the 18,000 police departments nationwide indicated that the number of violent crimes brought to the attention of law enforcement authorities decreased 21 percent between 1993 and 1998. Criminologists have provided numerous reasons for the decline in violent crime that incorporate demographic, economic, and policy-related explanations. Utilizing national data maintained by the Bureau of Justice Statistics (BJS), this article examines the extent to which changes in alcohol-involved violence may contribute to the declining rates of violent crime.

## Measuring the Extent to Which Alcohol and Other Drug Use IS Involved in Crime

Researchers face significant limitations in measuring the role of alcohol use in criminal behavior, because most alcohol consumption does not result in crime. In addition, nonoffending behavior is not typically measured; therefore, limited statistical information exists on which to estimate the likelihood that a person will commit a criminal act during or following alcohol consumption. This article examines two major sources of information on alcohol’s involvement in crime: (1) victim surveys and (2) offender surveys. A study reported by BJS in 1998, which used both of these resources, found that they yielded similar estimates regarding the involvement of alcohol and other drug use in crime. The investigators evaluated victim, offender, and law enforcement data from 1992 through 1995 and estimated that offenders had used either alcohol alone or alcohol with other drugs in approximately 37 percent of violent victimizations in which victims were able to describe substance use by the offenders (Greenfeld 1998). The study also found that in regard to violent offenders, 41 percent on probation, 41 percent in local jails, and 38 percent in State prisons reported that they had been using alcohol when they committed their offenses (Greenfeld 1998).

### Victims’ Perceptions of Offenders’ AOD Use

Most knowledge about the incidence and prevalence of violence derives from the National Crime Victimization Survey (NCVS), an ongoing survey of U.S. households conducted since 1973 by the BJS. The NCVS gathers information about exposure to and consequences of crime among the general U.S. population. Researchers use a nationally representative sample of approximately 50,000 U.S. households. All household members age 12 and older are interviewed twice per year and are asked about any crimes that they may have experienced during the preceding 6 months. More than 200,000 interviews are conducted each year, and approximately 7,000 respondents report having been victims of violent crime—such as rape, sexual assault, robbery, aggravated assault, and simple assault—or victims of attempted violence.

In 1986 researchers added new items to the NCVS in order to gain information about the following: victims’ perceptions of alcohol and other drug use by offenders, ways in which victims attempted to protect themselves, and victims’ descriptions of the criminal justice system’s response. Questions on alcohol and other drug use were only asked of victims of violence, because personal contact between victims and offenders was essential for collecting data on victims’ perceptions (Whitaker 1989). Estimates derived from the NCVS indicated that about 70 percent of victims of violence were consistently able to describe whether their offenders had been drinking or using other drugs.

The first report to reflect the new information, published in 1989, indicated that among victims of violence who could determine whether their offenders had been using alcohol, only slightly less than one-half (49 percent) of the victims believed that the offenders had used alcohol. According to the victims’ reports, male offenders were more likely than were female offenders to have been drinking, white offenders were more likely than were black offenders to have been drinking, and older offenders were more likely than were younger offenders to have been drinking. No significant differences were found in alcohol use between offenders who were strangers and offenders who were not strangers (Whitaker 1989).

Between 1993 and 1998, the number of violent victimizations experienced by the public dropped about 23 percent, from just over 10.5 million to about 8.1 million (see table 1). Based on victims’ self-reports, the number of violent crimes in which the offenders were perceived to be using only alcohol decreased 34 percent between 1993 and 1998, whereas the number of violent offenses in which the offenders were believed to be using only other drugs actually increased 19 percent. The number of violent victimizations in which the offenders were not believed to be using alcohol or other drugs decreased 22 percent.

In 1993 victims ascribed alcohol use to offenders in nearly 2.1 million violent crime incidents; in 1998 an estimated 1.4 million violent crimes were considered alcohol-involved based on the victims’ perceptions. Whereas the number of violent crimes experienced by all victims decreased by approximately 2.4 million during that period, the number of alcohol-involved crimes decreased by about 700,000.

Victim surveys also suggest that when a violent offender is suspected of substance use, alcohol use is more often suspected than is other drug use. Based on the data from victims of violence who were interviewed between 1993 and 1998 and who could describe their offenders’ use of alcohol and other drugs, an estimated 26 percent of the offenders were using only alcohol; about 8 percent were using only illicit drugs; about 7 percent were using both alcohol and other drugs; about 2 percent were using a substance not known to the victim; and the remaining offenders, totaling about 58 percent, were not believed to have been using any substance at the time of their offenses (see table 2).

Substance use appears to be linked more often in intimate partner violence than in violence between strangers. Between 1993 and 1998, nearly three out of four victims who suffered violence by an intimate partner (i.e., a current or former spouse, boyfriend, or girlfriend) reported that alcohol or other drug use had been present. Among spouse victims, 2 out of 3 incidents were reported to have involved an offender who had been drinking, and 8 out of 10 incidents involved alcohol, other drugs, or both. Among female victims of intimate partner violence, no differences were found in perceived alcohol or other drug use by race; approximately 6 out of 10 of black, white, and Hispanic women reported that the offender had been drinking or had been both drinking and using other drugs (see table 3). Victims perceived the offender to be using either alcohol alone or both alcohol and other drugs in only about 29 percent of violent incidents between strangers, however (see table 2).

Offenders’ substance use (as perceived by victims) also varied by type of crime. Based on victim reports, drinking offenders committed more than one-third of the rapes and sexual assaults among victims age 12 and older and more than one-fourth of the aggravated and simple assaults. Among the alcohol-involved rapes and sexual assaults, the offender was perceived as using alcohol in combination with other drugs in 18 percent of the cases. Other drug use also was perceived by victims in 33 percent of the alcohol-involved robberies, 26 percent of the alcohol-involved aggravated assaults, and 16 percent of the alcohol-involved simple assaults.

Table 4 illustrates the relationships between types of offender substance use and types of offenses. Among those cases in which the victim reported that the offender was using only alcohol (i.e., no other drugs) at the time of the offense, nearly two-thirds of the offenses were simple assaults. Robbery accounted for about 6 percent of the offenses by offenders reported to have used only alcohol but accounted for about 19 percent of the incidents in which the offenders were perceived to have been using other drugs.

### Characteristics of Victimizations Involving Alcohol

From 1993 through 1998, nearly one-half of the violent victimizations in which the victim reported alcohol use by the offender (i.e., alcohol-involved incidents) occurred in a residence, and more than 20 percent occurred in the victim’s home. One of seven alcohol-involved incidents occurred near or at the victim’s workplace. More than one-third of the incidents involving alcohol occurred in a public place (e.g., commercial areas, parking lots, schools, or parks), with the most common location being an open area, such as in a park or on the street.

Almost one-half of the violent victimizations involving alcohol use by the offender occurred between 6 p.m. and 12 a.m., and slightly more than one-fourth of these incidents occurred between 12 a.m. and 6 a.m. The fewest incidents occurred between 6 a.m. and 12 p.m.

Overall, about 26 percent of all violent incidents from 1993 through 1998 involved the use of a weapon, 36 percent involved the use of hands and feet only, and 38 percent involved neither a weapon nor hands and feet. Alcohol-involved offenders, as described by victims, were as likely as violent offenders in general to have not used a weapon or to have used their hands and feet only. Slightly more than one-fourth of the alcohol-involved incidents involved a weapon. Among all alcohol-involved incidents, victims reported firearm use in 9 percent of the cases, whereas firearms were used in 11 percent of violent incidents that were not alcohol related.

Approximately one-third of the alcohol-involved victimizations resulted in an injury to the victim. About one in five victims of violence who perceived the offender to have been using alcohol at the time of the offense (i.e., approximately 400,000 victims per year) suffered a financial loss attributable to medical expenses, broken or stolen property, or lost wages––equaling an annual loss of $400 million (see table 5).

## Alcohol Use Among Convicted Offenders

In addition to victims’ reports of offender substance use, researchers also draw on surveys of offenders concerning their use of alcohol and other drugs in general as well as in relation to a particular offense. The BJS periodically conducts surveys among the Nation’s population of offenders to learn more about their backgrounds.^1^ Representative samples of probationers and offenders in local jails and in State and Federal prisons are interviewed about their criminal histories, family backgrounds, and numerous elements of their offenses. Among the topics of interest is their use of alcohol, both in the past and at the time of the offense. Such information is not typically available from official records; these surveys provide the only uniform national description of offenders’ use of alcohol. Changes in the extent to which offenders’ self-report alcohol use as a factor in their offenses may be an important indicator of changes in offending.

On an average day in 1998, an estimated 5.7 million convicted offenders were under the supervision of criminal justice authorities. Based on nationally representative sample surveys conducted among probationers (Survey of Adults on Probation, 1996), jail inmates (Survey of Inmates in Local Jails, 1996), and prisoners in custody in State and Federal prisons (Surveys of Inmates in State and Federal Correctional Facilities, 1997), nearly 38 percent of those offenders were drinking at the time of the offenses for which they were convicted.^2^ (For information regarding sources of data on alcohol and crime, see sidebar, p. 30.) This estimate translates into more than 2 million convicted offenders nationwide on an average day—1.4 million on probation, 100,000 in local jails, 460,000 in State and Federal prisons, and more than 200,000 under parole supervision—for whom alcohol might have been a factor in their crimes (see figure).

**Sources of Data on Alcohol and Crime**

***National Crime Victimization Survey***

The National Crime Victimization Survey (NCVS) is one of two statistical series maintained by the Department of Justice to learn the extent to which crime is occurring. The NCVS, which gathers data on criminal victimization from a national sample of household respondents, provides annual estimates of crimes experienced by the public without regard to whether a law enforcement agency was called about the crime. Initiated in 1972, the NCVS was designed to complement what is known about crimes reported to local law enforcement agencies under the Federal Bureau of Investigation’s (FBI’s) annual compilation known as the Uniform Crime Reports. The NCVS gathers information about crime and its consequences from a nationally representative sample of U.S. residents age 12 and older about any crimes they may have experienced. For personal-contact crimes, the survey determines who the perpetrator was. Asking the victim about his or her relationship to the offender is critical to determining whether the crime occurred between intimates.

In the latter half of the 1980s, the Bureau of Justice Statistics (BJS), together with the Committee on Law and Justice of the American Statistical Association, sought to improve the NCVS components to enhance the measurement of certain crimes, including rape, sexual assault, and intimate and family violence. The new questions and revised procedures were phased in from January 1992 through June 1993 in one-half of the sampled households. Since July 1993 the redesigned methods have been used for the entire national sample.

***Uniform Crime Reporting Program***

The Uniform Crime Reporting Program (UCR) of the FBI provides another opportunity to examine the issue of intimate violence. The summary-based component of the UCR, launched 70 years ago, gathers aggregate data on eight categories of crime from law enforcement agencies nationwide. Although the summary UCR provides detailed information on people arrested for driving while under the influence, it does not provide any information necessary to identify either violent crimes or arrests for crimes involving alcohol other than driving while intoxicated (DWI). Such data are available, however, from the incident-based component of the UCR, entitled the National Incident-Based Reporting Program (NIBRS).

***National Incident-Based Reporting Program***

NIBRS represents the next generation of crime data from law enforcement agencies. Rather than being restricted to a group of 8 index crimes that the summary-based program uses, NIBRS obtains information on 57 types of crimes. The information collected on each violent crime incident includes victim-offender demographics, victim-offender relationship, time and place of occurrence, weapon use, and victim injuries. An important contribution of NIBRS is that investigating officers are asked to record their perceptions of whether alcohol was a factor in the incident. As of the end of 1998, the FBI had certified 18 State-level programs for NIBRS participation, and an additional 16 State agencies as well as a number of local law enforcement agencies had prepared test data for the FBI to evaluate in regard to status of implementation. NIBRS data collection, as the next generation of law enforcement data, will play a significant role in the future in improving our knowledge of violence with special regard for such concerns as intimate violence, family violence, and domestic violence as well as the role alcohol may play in these kinds of police-reported incidents.

***Surveys of Probationers as well as Jail and Prison Inmates***

BJS also conducts national surveys of people under probation supervision as well as people confined in local jails and State and Federal prisons. These nationally representative surveys are the principal source of information on people serving time following a conviction: their backgrounds, their prior criminal histories, and the circumstances surrounding the offenses for which they had been incarcerated. Both jail and prison surveys obtain from each violent offender details about the offender’s relationship to the victim and how the crime was carried out. All three surveys incorporate detailed questions regarding alcohol use and abuse, both before the crime and at the time the crime was committed. In addition, a number of questions are devoted to treatment and the types of treatments received.

***Censuses of Prisons and Jails***

BJS carries out facility-level data collection among each of the 1,500 State and Federal prisons and the 3,300 local jails. These surveys gather detailed information on the operations of each facility, including capacity, staffing, programs, court orders, and special functions or services provided to inmates.

***Fatal Accident Reporting System***

Since 1975 the National Highway Traffic Safety Administration of the U.S. Department of Transportation has maintained the annual Fatal Accident Reporting System (FARS), which obtains accident-level data on each motor vehicle crash involving a fatality. FARS utilizes State agencies under contract to complete a standardized form on each fatal accident, including information on weather and road conditions, vehicle type, number of passengers and fatalities, manner of the crash, whether a drinking or drug-using driver was involved, and a specific measurement of blood alcohol concentration (BAC) (i.e., grams of alcohol per deciliter of blood) if applicable.

—Lawrence A. Greenfeld and Maureen A. Henneberg

Offenders on probation and those in jail or State prison reported similar rates of drinking at the time of their offenses. Offenders convicted of public order offenses, such as drinking and driving, weapons offenses, and commercial vice (e.g., prostitution, gambling, or pornography) were the most likely to report alcohol use at the time of the offense (see table 6). Offenders convicted of violent crimes also reported high rates of drinking. For example, more than 40 percent of murderers in jail or State prison reported that they had been drinking at the time of their offenses, and nearly one-half of those convicted of assault and sentenced to probation had been drinking when the offenses occurred.

Offenders on probation, in local jails, and in State prisons were about equally likely to have been drinking at the time of their offenses. Beer was the most commonly used alcoholic beverage: 30 percent of probationers, 32 percent of jail inmates, and 28 percent of State prisoners said that they had been drinking beer or both beer and liquor prior to committing the offenses. Consumption of wine alone was comparatively rare among the surveyed offenders (see table 7).

### Alcohol Use Among Offenders on Probation

Male probationers were more likely than female probationers to report alcohol use at the time of their offenses (41 vs. 25 percent) (see table 8). For probationers who had been convicted of public order crimes, nearly two-thirds of the women and three-fourths of the men had been drinking at the time of their offenses.

In addition to alcohol use at the time of the violent offenses, the survey gathered information about the probationers’ alcohol use during other times in their lives. For example, findings indicated the following:

- About one-half of all probationers reported that they had driven a vehicle while under the influence of alcohol.
- About one-half of all probationers had engaged in arguments with their families or friends while drinking.
- About one-third of probationers had gotten into a physical fight with someone after drinking.
- More than one-third of probationers reported that they had consumed the equivalent of one-fifth of liquor in 1 day.
- About 1 in 12 probationers said that they had lost a job because of drinking.

### Alcohol Use Among Inmates of Local Jails

As was found among probationers, convicted males in local jails were more likely than convicted females to report alcohol use at the time of their offenses, although the disparity between the genders was smaller (see table 9). For every type of offense committed, except public order offenses, women in jail were more likely to report having used alcohol at the time of the offenses than were women on probation.

About 60 percent of convicted jail inmates said that they had been drinking on a regular basis (i.e., drinking alcohol more than once per week for more than 1 month) during the year before the offense for which they were serving time. Nearly two-thirds of those inmates, regardless of whether they drank daily or less often, reported having been previously in a treatment program for alcohol dependence. About one-third of all convicted inmates in local jails described themselves as having been daily drinkers at the time of their offenses. Among these daily drinkers, about two out of three said that they had previously received some form of treatment for alcohol dependency. Among those who described themselves as drinking less often, about two-thirds reported prior alcoholism treatment participation, most often in an inpatient program.

### Alcohol Use Among Inmates in State Prisons

The alcohol consumption patterns of State prisoners differed markedly from those of jail inmates^3^ and probationers. Although the prevalence of drinking was lower, the estimated levels of intoxication at the time of the offenses were higher. Overall, State prison inmates reported having consumed an average of more than 6 ounces of alcohol prior to their offenses, the equivalent of about 2 six packs of beer or 2 quarts of wine. The average time spent drinking before committing the crime was about 4 hours.

The inmate survey also provided an opportunity to query people involved in personal-contact crimes about their perceptions of the victims’ alcohol use at the time of the crime. Manslaughter offenses and offenses directed against a spouse or intimate partner were the most likely offenses to have involved alcohol. The survey indicated that for offenders in State prisons on manslaughter convictions, alcohol use (by the offender, victim, or both) was a factor in about two-thirds of the offenses (see table 10). In addition, the survey found that alcohol use by both the victim and the offender was most common in violent offenses directed at spouses and other intimate partners.

Nearly 30 percent of State prisoners described themselves as daily drinkers preceding their incarceration (see table 11). By type of offense, limited variation existed in the percentage of offenders who described themselves as daily drinkers, although those serving time for drug offenses were slightly less likely to report daily drinking. Nearly one-half of all drinking-and-driving offenders in State prisons described themselves as daily drinkers. Overall, the average age of drinking initiation was lowest among daily drinkers (i.e., 14.5 years).

The prisoner survey indicated that for more than one-half of the murder cases, either the offender, the victim, or both parties had used alcohol at the time of the murder (see table 12). Those who murdered intimate partners reported drinking for the longest period prior to the offense.

Male State prisoners were more likely than were female prisoners to report that they were under the influence of alcohol at the time of the offense (38 vs. 29 percent) and to report having ever engaged in binge drinking^4^ (42 vs. 30 percent). However, about one-fourth of both men and women were found to have alcohol problems (i.e., they had three or more positive responses to the CAGE questionnaire [Ewing 1984], a screening instrument used to detect alcohol abuse and dependence) (see table 13) (Mumola 1999).

Among State inmates, non-Hispanic whites were most likely to report binge drinking, being under the influence of alcohol at the time of the offense, and alcohol problems, followed by Hispanics and then non-Hispanic blacks. About 53 percent of non-Hispanic whites reported having ever engaged in binge drinking, 43 percent reported committing their offenses under the influence of alcohol, and 33 percent met the CAGE criteria for possible alcohol abuse or dependence. The prisoners’ reports of these three measures were not related to age; among those State prisoners in the age groups between 25 and 54, similar percentages were reported on all three measures (see table 13) (Mumola 1999).

### Trends in Alcohol Use Among Convicted Offenders

Comparing rates of offenders’ self-reported alcohol use from year to year reveals opposite trends for local jail inmates and prison inmates. From 1983 to 1996, self-reported alcohol use prior to the offense decreased from 48 to 40 percent among all local jail inmates and from 54 to 41 percent among inmates convicted of violent offenses. In contrast, the number of violent offenders in State prisons who reported alcohol use prior to their offenses increased 71 percent from 1991 to 1997. (The number of violent offenders in State prisons increased 51 percent during this period.) At the same time, the number of violent offenders in State prisons who were not drinking when their offenses occurred increased 39 percent.

## The Role of Alcohol Use in Declining Rates of Violence

Consistent with the decrease in jail inmates’ self-reported alcohol use, victim surveys suggest a decrease in violent crime and alcohol-involved violence. The most recent victimization data just made available by BJS indicate that the trend in more rapid declines for alcohol-involved offenses is continuing. Between 1993 and 1998, violent crime victimization declined by one-fourth. Based on victim reports from 1998, alcohol use by the offender was a factor in about 1.9 million violent victimizations, compared with 2.7 million in 1993. On a per capita basis, this translates into a decrease in the rate of alcohol-involved violent crime from more than 12 incidents per 1,000 to just over 8 incidents per 1,000, a 34-percent decrease. In contrast, the rate of non-alcohol-involved violence decreased 24 percent during the same period.

About 2.4 million fewer violent crimes occurred in 1998 than in 1993, including more than 800,000 fewer violent crimes in which the offender was perceived to have been using alcohol. We would estimate that more than 30 percent of the drop in violence during that period could be attributed to averted alcohol-involved crime.

The decrease in alcohol-involved violence suggested by victim surveys is reflected in other measures that indicate a decrease in alcohol use and related problems. For example, the proportion of respondents to the National Household Survey on Drug Abuse who described themselves as current drinkers (i.e., persons who reported the consumption of alcohol within the month preceding the survey) decreased for every age group from 1985 to 1997 (Office of Applied Studies 2000). Among 12- to 17-year-olds, this measure decreased from 41 to 21 percent; among 18- to 25-year-olds, the decrease was from 70 to 58 percent; among 26- to 34-year-olds, 71 to 60 percent; and among people age 35 and older, 58 to 53 percent. A smaller percentage of each age group therefore described themselves as current drinkers during the 1990s, when crime rates were falling, compared with the 1980s, a period in which crime rates were rising.

Consumption of alcohol among young people, in particular, sharply decreased. Nearly 85 percent of high school seniors in 1986 reported using alcohol during the preceding year, compared with 74 percent in 1998 who reported drinking during the prior year. When asked whether they had consumed alcohol during the previous 30 days, high school seniors’ responses reflected the same pattern—a decrease from 65 percent in 1986 to 52 percent in 1998, again illustrating the point that during periods of higher consumption, crime rates were also higher, whereas periods of lower consumption were marked by lower crime rates. Recent surveys of college students showed similar decreases in alcohol consumption both in the past year and in the past 30 days during the 1990s.

During the same period, per capita consumption of alcoholic beverages (i.e., beer, wine, and distilled spirits) decreased more than 4 percent, from 40.7 gallons per person annually in 1985 to 38.9 gallons in 1997, according to U.S. Department of Agriculture data (U.S. Census Bureau 1999). Data on taxable production of alcoholic beverages compiled by the Bureau of Alcohol, Tobacco, and Firearms show that production in the late 1990s also was well below levels reported in the early 1980s. Compared with 1980, production of distilled spirits dropped 56 percent, whiskey production dropped 21 percent, and wine production declined 58 percent by the latter half of the 1990s (U.S. Census Bureau 1999).

Alcohol-involved traffic fatalities also have decreased in recent years and have been a major factor in the overall decline in automobile fatalities. Between 1982 and 1989, the average annual number of automobile fatalities was 44,970—of which an average of 23,625 involved alcohol. Between 1992 and 1999, the annual average number of fatalities decreased 8.5 percent to 41,136, and an annual average of 16,785 fatalities were attributed to alcohol-involved crashes, a decline of 29 percent (National Highway Traffic Safety Administration 2000). The decline in alcohol-involved traffic deaths in the 1990s was more than three times the rate of decrease in all traffic fatalities.

Similarly, between 1993 and 1998, overall arrests by police increased 3.5 percent (from 14,036,300 to 14,528,300), whereas drinking and driving arrests decreased 8 percent (from 1,524,800 to 1,402,800). In other words, both alcohol-involved motor vehicle fatalities and criminal arrests for driving under the influence of alcohol have declined sharply during the recent past.

Although establishing a clear relationship between reductions in alcohol use and declines in violent crime victimization is difficult, some evidence exists to support this association. In a study that evaluated the rates of alcohol consumption and homicide since Prohibition, Parker (1998) concluded that for most of the 62-year period, strong positive correlations existed between changes in alcohol consumption and changes in homicide rates.

Inmates in local jails and those on probation are the most likely members of the correctional population to reflect changes in alcohol consumption. Drinking and driving and other types of alcohol-involved offenses are highest among these populations (see table 6). Unfortunately, trend data are not available for the probation population and are only available for the jail population based on the national sample surveys conducted in 1983, 1989, and 1995. As noted previously, self-reported alcohol use prior to the offenses among violent offenders in local jails decreased from 1983 to 1996. This decline is consistent with a reduction in alcohol-involved violence indicated by the victim survey results and other measures indicating reduced alcohol-involved problems.

The rapid increase in the size of the alcohol-involved offender population in State prisons is inconsistent with the trend observed for local jail inmates. Survey results from 1991 indicate that 32 percent of State prisoners had been using alcohol at the time of their offenses, compared with 37 percent in 1997. Between 1990 and 1998, violent offenders were the fastest-growing segment of the State prison population, accounting for 53 percent of the total increase nationally. This growth was largely the result of increased lengths of stay (Beck 2000). Among court-committed prison admissions, public order offenders (e.g., weapons offenses, commercial vice, and driving while intoxicated [DWI]) were the fastest growing component, expanding at nearly six times the rate of increase observed for all prison admissions from courts. In 1990 a total of 26,000 of the 323,069 court-committed offenders were public order offenders, compared with 37,500 of the 347,270 court-committed offenders in 1998. Therefore, the number of public order offenders committed to prison by courts increased 44.2 percent, whereas the number of total court-committed offenders increased 7.5 percent (Beck 2000).

These two groups of offenders, violent offenders and public order offenders, were substantially more likely than other groups of offenders (e.g., property offenders [those convicted of burglary, larceny, or fraud, for example] and drug offenders) to have been consuming alcohol at the time of their offenses. Between 1990 and 1997, State prison populations grew 57 percent. During the same period, the number of drinking-and-driving offenders, a major segment of the public order population in State prisons, increased by 83 percent.

When analyzed together, these data suggest that reduced consumption of alcohol is co-occurring with a reduction in violent crime as well as many other positive outcomes, including reduced traffic fatalities and declining rates of arrest for alcohol-involved traffic offenses. For victims of violence in 1998, this translates into about 700,000 fewer violent crimes than those experienced just 5 years earlier, which had been attributed to offenders who had been drinking alcohol. At the beginning of the decade, about one-half of all traffic deaths resulted from alcohol-involved crashes; at the end of the decade, nearly 7,000 fewer alcohol-involved traffic fatalities were recorded, whereas the number of licensed drivers and the number of vehicle miles traveled increased.

Although the reductions in alcohol consumption have been accompanied by reductions in alcohol-involved violence, much additional research still needs to establish the extent to which the crime reductions can be directly attributed to decreases in alcohol use. BJS will initiate new self-report surveys of local jail inmates in 2001 and of State and Federal prisoners in 2002, which will include more detailed items on alcohol use and treatment program participation. In addition, the NCVS will experiment with items intended to learn more about victim use of alcohol at the time of the offense. In particular, researchers must learn more about domestic and spousal violence and the role of alcohol in such incidents. Finally, BJS will undertake experiments in selected cities to evaluate the effect of moving from the current Supplementary Homicide Reporting Program, which provides no data to disaggregate alcohol-involved homicide incidents, to the National Incident-Based Reporting System, which will permit investigating officers to indicate whether a homicide was determined to be alcohol related. These evolving efforts will permit more substantial analyses of the relationship between alcohol use and abuse and crime incidence in the future.

## Figures and Tables

**Figure:**
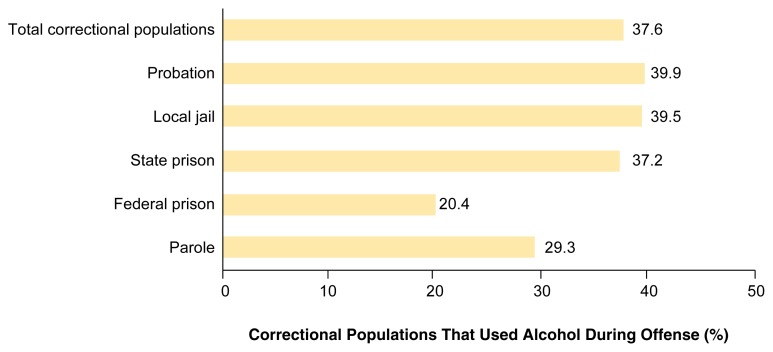
Percentages of correctional populations estimated to have been using alcohol at the time of their conviction offenses. The correctional populations are categorized by correctional status (e.g., probation, State prison, or parole) during 1998. SOURCE: Bureau of Justice Statistics surveys of correctional populations.

**Table 1:** Number of Violent Victimizations by Perceived Offender Substance Use, 1993 and 1998

Offender Substance Use	Victims’ Perceptions of Offender Substance Use		

	1993 (*n*)	1998 (*n*)	Change in Perception From 1993 to 1998 (%)
All violent victimizations	10,531,582	8,116,238	−23
Alcohol	2,069,457	1,365,903	−34
Illicit drugs	443,426	526,522	19
Both alcohol and illicit drugs	614,410	497,930	−19
No alcohol or illicit drugs	4,247,235	3,323,789	−22
Victim did not know	3,157,054	2,402,094	−24

SOURCE: Bureau of Justice Statistics’ National Crime Victimization Survey, 1993 and 1998.

**Table 2:** Offender Substance Use at the Time of Offense, by Victim-Offender Relationship, 1993–1998

Victim-Offender Relationship	Offender Substance Use*(%)				

	Total	Alcohol	Other Drugs	Alcohol and/or Other Drugs	No Substance Use
All victims of violence	100	26	8	9	58
Intimate partner**	100	51	10	12	28
Other family member	100	38	16	13	34
Acquaintance	100	26	10	11	53
Stranger	100	22	6	7	65

* Offender substance use is based on reports from victims who were able to describe whether an offender had been using alcohol and/or other drugs at the time of the offense.

** Includes current or former spouse, boyfriend, or girlfriend. Substance use is more likely to be a factor in intimate partner violence compared with violence between strangers.

NOTE: Due to rounding, the percentages within the categories may not total 100 percent.

SOURCE: National Crime Victimization Survey, 1993–1998.

**Table 3:** Rates of Substance Use Among Violent Offenders as Reported by Female Victims, by Victims’ Race/Ethnicity, 1993–1998*

Offender Substance Use**	Female Victims’ Race/Ethnicity (%)			

	All	White	Black	Hispanic
Alcohol only	52	53	49	52
Other drugs only	10	11	8	9
Both	11	11	8	5
Either	2	2	3	1
Neither	25	23	31	33

* Rates reflect offender substance use in cases in which the victim was a female and the victim and offender were intimate partners.

** Offender substance use is based on reports from victims who were able to describe whether an offender had been using alcohol and/or other drugs at the time of the offense.

NOTE: Due to rounding, the percentages within the categories may not total 100 percent.

SOURCE: National Crime Victimization Survey, 1993–1998.

**Table 4:** Offender Substance Use at the Time of Offense, by Type of Offense, 1993–1998

Type of Violent Offense	Offender Substance Use* (%)			

	Alcohol Only	Other Drugs Only	Alcohol and Other Drugs	No Substance Use
Total	100	100	100	100
Rape/sexual assault	7	3	5	3
Robbery	6	19	11	17
Aggravated assault	23	26	31	23
Simple assault	65	52	52	57

* Offender substance use is based on reports from victims who were able to describe whether an offender had been using alcohol and/or other drugs at the time of the offense.

NOTE: Due to rounding, the percentages within the categories may not total 100 percent.

SOURCE: National Crime Victimization Survey, 1993–1998.

**Table 5:** Estimated Annual Costs to Victims of Alcohol-Involved Violence by Type of Expense, 1993–1998*

Type of Expense/Loss	Average Loss per Victim	Estimated Total Annual Loss
Total	$1,016	$401,800,000
Medical expenses	2,033	230,400,000
Cash loss	248	10,500,000
Property		
Loss	604	49,500,000
Repair	267	32,300,000
Replacement	310	23,000,000
Lost pay from		
Injury	711	40,200,000
Other causes	428	15,900,000

* Victims of alcohol-involved violence lost a total of $400 million each year from 1993 to 1998.

SOURCE: National Crime Victimization Survey, 1993–1998.

**Table 6:** Convicted Offenders Who Reported Drinking at the Time of Offense, by Offense Type

Type of Offense	Current Offender Status (%)			

	Probation	Local Jail Inmate	State Prison Inmate	Federal Prison Inmate
All offenses	39.9	39.5	37.2	20.4
Violent offense	40.7	40.6	41.7	24.5
Murder	*	43.7	44.6	38.7
Rape/sexual assault	31.8	31.5	40.0	32.3
Robbery	*	37.6	37.4	18.0
Assault	45.5	45.4	45.1	46.0
Property offense	18.5	32.8	34.5	15.6
Burglary	38.5	38.2	37.2	*
Larceny	16.3	31.6	33.7	*
Fraud	9.7	21.6	25.2	10.4
Drug offense	16.3	28.8	27.4	19.8
Possession	14.4	28.6	29.6	21.3
Trafficking	16.2	28.4	25.5	19.4
Public order offense**	75.1	56.0	43.2	20.6

* Too few cases for estimate to be made.

** Offenders convicted of a public order offense, such as drinking and driving, weapons, and commercial vice (e.g., prostitution, gambling, or pornography), were the most likely to report alcohol use at the time of the offense.

SOURCE: Data for this table are drawn from the 1996 Survey of Adults on Probation, the 1996 Survey of Inmates in Local Jails, and the 1997 Surveys of Inmates in State and Federal Correctional Facilities.

**Table 7:** Alcoholic Beverages Consumed by Convicted Offenders Who Were Drinking at the Time of Offense, From Selected Surveys

Beverage Consumed at Time of Offense	Current Offender Status (%)		

	Probation	Local Jail Inmate	State Prison Inmate
Beer	20	20	15
Liquor	6	4	6
Beer and liquor	10	12	13
Other combinations	2	4	2
None	62	60	64

SOURCE: Data for this table are drawn from the 1996 Survey of Adults on Probation, the 1996 Survey of Inmates in Local Jails, and the 1997 Surveys of Inmates in State and Federal Correctional Facilities.

**Table 8:** Percentage of Adult Probationers Who Reported Using Alcohol at the Time of Offense, by Type of Offense and Gender

	Adult Probationers Who Reported Drinking at Time of Offense (%)	

	Male*	Female
Total	41	25
Violent offense	38	28
Property offense	21	6
Drug offense	17	12
Public order offense	75	62

* Male probationers were more likely than female probationers to report drinking at the time of the offense.

SOURCE: 1996 Survey of Adults on Probation.

**Table 9:** Percentage of Inmates in Local Jails Who Reported Using Alcohol at the Time of Offense, by Type of Offense and Gender, 1996

Type of Offense	Inmates in Local Jails Who Reported Drinking at Time of Offense (%)	

	Male*	Female
Total	41	29
Violent offense	41	35
Property offense	35	16
Drug offense	29	27
Public order offense	57	47

* Male inmates were more likely than female inmates to report drinking at the time of the offense.

SOURCE: Survey of Inmates in Local Jails, 1996.

**Table 10:** Alcohol Use at the Time of Offense by Offender, Victim, or Both, as Reported by State Prison Inmates, 1997

	Alcohol Use at Time of Offense (%)			

	Offender Only	Victim Only	Both	Neither
**All Offenders**	28	9	13	51
**Offense**				
Murder	23	12	20	45
Manslaughter	24	17	27	32
Rape/sexual assault	31	2	8	59
Robbery	31	6	5	57
Assault	24	13	20	43
**Victim-offender relationship**				
Spouse/intimate partner	21	10	23	46
Family member	30	3	4	63
Acquaintance	24	12	19	45
Stranger	31	9	10	51

NOTE: Due to rounding, the percentages within the categories may not total 100 percent.

SOURCE: Survey of Inmates of State Correctional Facilities, 1997.

**Table 11:** State Prisoners’ Self-Reported Alcohol Use Before Incarceration by Most Serious Offense, 1997

Most Serious Offenses Committed	State Prison Inmates’ Self-Reported Alcohol Use (%)				

	Total	Non-Drinkers	Daily Drinkers	Weekly Drinkers	Drink Less Often Than Weekly
All	100	35	28	23	14
Violent offense	100	34	29	23	14
Murder	100	32	31	22	15
Manslaughter	100	32	26	25	18
Sexual assault	100	36	26	23	15
Robbery	100	36	30	22	12
Assault	100	31	28	26	16
Property offense	100	35	30	22	14
Drug offense	100	39	25	22	14
Public order offense*	100	36	28	23	13
DWI	100	8	49	34	9
Average age of drinking initiation (years)	NA	16.6	14.5	15.8	16.1

* Includes other offenses not classified.

DWI = driving while intoxicated; NA = not applicable.

NOTE: Due to rounding, the percentages within the categories may not total 100 percent.

SOURCE: Survey of Inmates of State Correctional Facilities, 1997.

**Table 12:** Alcohol Use by Murder Victims and Offenders as Reported by State Prisoners, 1997

Drinking at Time of Murder	Relationship of Victims of Murderers in State Prisons (%)				

	All	Intimate Partner	Family Member	Acquaintance	Stranger
Total	100	100	100	100	100
Murderer	23	15	32	23	25
Victim	13	14	9	13	12
Both	21	21	11	24	18
Neither	44	50	48	40	46

NOTE: Due to rounding, the percentages within the categories may not total 100 percent.

SOURCE: Survey of Inmates of State Correctional Facilities, 1997.

**Table 13:** Self-Reported Alcohol Use and Abuse Among State Prisoners, 1997

Inmates’ Personal Characteristics	State Prison Inmates’ Self-Reported Alcohol Use			

	Estimated Number of Prisoners (*n*)	Ever Had a Binge-Drinking* Experience (%)	Committed Offense Under Alcohol Influence (%)	Had Three or More Positive CAGE** Responses (%)
All State prisoners	1,059,607	41.0	37.2	24.4
Male	993,365	41.8	37.7	24.5
Female	66,242	29.9	29.1	23.4
Race/Hispanic origin				
Non-Hispanic white	352,864	53.5	42.7	33.5
Non-Hispanic black	492,676	31.9	33.0	18.6
Hispanic	179,998	39.9	36.7	22.0
Other	34,069	49.6	41.7	27.7
Age (years)				
24 or younger	209,343	40.2	30.7	15.8
25–34	404,034	42.3	37.7	24.8
35–44	311,999	42.3	41.3	28.6
45–54	103,470	37.4	37.7	28.5
55 or older	30,761	29.3	30.2	22.5

* Binge drinking is defined as having consumed in 1 day as much as one-fifth of liquor, equivalent to 20 drinks, 3 bottles of wine, or 3 six-packs of beer.

** The CAGE questionnaire (Ewing 1984) is a screening instrument for detecting a person’s history of alcohol abuse or dependence.

SOURCE: Survey of Inmates of State Correctional Facilities, 1997.

## Footnotes

1 Unless otherwise noted, surveys cited in this article were conducted by the Bureau of Justice Statistics (BJS), U.S. Department of Justice. Additional information about these surveys can be obtained from the BJS.

2 About 4 in 10 murderers in State prison reported that they had been consuming alcohol at the time of the offense. An additional 10 percent reported that only the victim had been drinking alcohol at the time of the homicide.

3 Jail inmates are those offenders sentenced to serve time in a locally operated correctional facility, generally with a sentence of 1 year or less. State prisoners, by contrast, are those offenders sentenced to a State-operated correctional facility with sentences generally exceeding 1 year.

4 Binge drinking is defined as having consumed in 1 day as much as one-fifth of liquor, equivalent to 20 drinks, 3 bottles of wine, or 3 six-packs of beer.
